# Activating Molybdenum Carbide Nanoparticle Catalysts under Mild Conditions Using Thermally Labile Ligands

**DOI:** 10.1021/acs.chemmater.2c02148

**Published:** 2022-09-22

**Authors:** Lanja R. Karadaghi, Anh T. To, Susan E. Habas, Frederick G. Baddour, Daniel A. Ruddy, Richard L. Brutchey

**Affiliations:** † Department of Chemistry, University of Southern California, Los Angeles, California 90089, United States; ‡ Catalytic Carbon Transformation and Scale-Up Center, National Renewable Energy Laboratory, Golden, Colorado 80401, United States

## Abstract

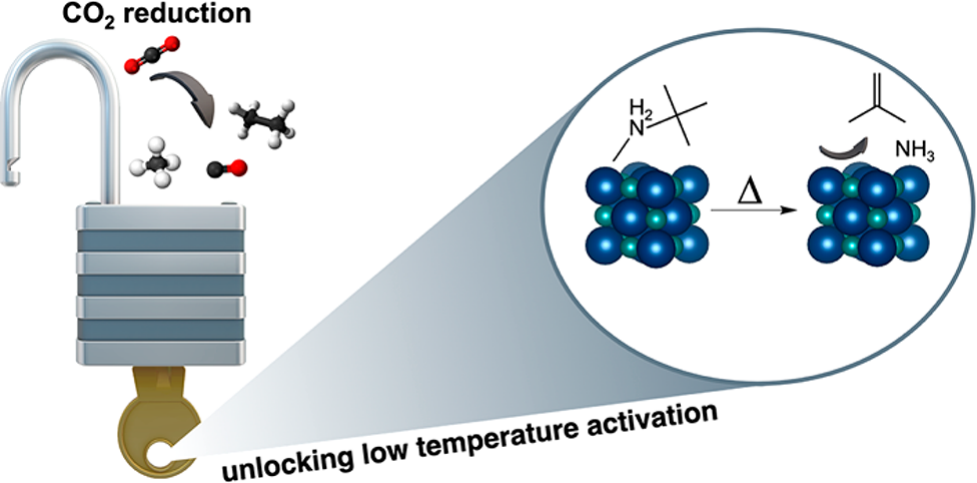

Transition-metal carbides are promising low-cost materials for various catalytic transformations due to their multifunctionality and noble-metal-like behavior. Nanostructuring transition-metal carbides offers advantages resulting from the large surface-area-to-volume ratios inherent in colloidal nanoparticle catalysts; however, a barrier for their utilization is removal of the long-chain aliphatic ligands on their surface to access active sites. Annealing procedures to remove these ligands require temperatures greater than the catalyst synthesis and catalytic reaction temperatures and may further result in coking or particle sintering that can reduce catalytic performance. One way to circumvent this problem is by replacing the long-chain aliphatic ligands with smaller ligands that can be easily removed through low-temperature thermolytic decomposition. Here, we present the exchange of native oleylamine ligands on colloidal α-MoC_1–*x*_ nanoparticles for thermally labile *tert*-butylamine ligands. Analyses of the ligand exchange reaction by solution ^1^H NMR spectroscopy, FT-IR spectroscopy, and thermogravimetric analysis–mass spectrometry (TGA-MS) confirm the displacement of 60% of the native oleylamine ligands for the thermally labile *tert*-butylamine, which can be removed with a mild activation step at 250 °C. Catalytic site densities were determined by carbon monoxide (CO) chemisorption, demonstrating that the mild thermal treatment at 250 °C activates ca. 25% of the total binding sites, while the native oleylamine-terminated MoC_1–*x*_ nanoparticles showed no available surface binding sites after this low-temperature treatment. The mild pretreatment at 250 °C also shows distinctly different initial activities and postinduction period selectivities in the CO_2_ hydrogenation reaction for the ligand exchanged MoC_1–*x*_ nanoparticle catalysts and the as-prepared material.

## Introduction

Transition-metal carbides (TMCs) have emerged as important catalytic materials because of their inherent multifunctionality that enables a wide range of transformations, including CO_2_ hydrogenation, ammonia synthesis, alkene hydrogenation, deoxygenation, and hydrodeoxygenation reactions.^1−5^ In addition to being low-cost, thermodynamically stable materials, TMCs also exhibit noble-metal-like behavior in terms of catalytic activity and electronic structure, and they resist corrosion because of their refractory nature.^6−8^ More recently, it has been established that TMC nanoparticle catalysts possess benefits when compared to their bulk counterparts because of the large surface-area-to-volume ratios that allow for an increase in exposed active sites.^9,10^ While TMC nanoparticles are inherently advantageous, the synthetic methods typically used to prepare them involve harsh reaction conditions (e.g., high-temperature carburization) that limit size control over the resulting catalysts.^11^ In 2020, we reported a mild solution-phase synthesis of phase-pure α-MoC_1–*x*_ nanoparticle catalysts through the thermolytic decomposition of Mo(CO)_6_ at relatively low synthesis temperatures (i.e., 290–320 °C). The α-MoC_1–*x*_ nanoparticles were evaluated for thermocatalytic CO_2_ hydrogenation and shown to exhibit an increased activity on a per-site basis compared to bulk α-MoC_1–*x*_.^5^ We further demonstrated that the α-MoC_1–*x*_ nanoparticle catalyst synthesis could be scaled up using continuous flow chemistry, while retaining the same catalytic performance as the nanoparticles prepared in the small-scale batch method.^12^

Although the large surface-area-to-volume ratio of nanoparticles is what drives the increased activity in these nanoparticle catalysts, the solution-phase syntheses of colloidal nanoparticles necessitates the use of long-chain, aliphatic ligands (e.g., C-18 oleylamine) to arrest particle growth and sterically stabilize the colloidal suspension.^13,14^ While these long-chain aliphatic ligands are necessary for synthesis and colloidal stabilization, they create steric barriers around the nanoparticles themselves, thereby blocking active sites at the surface.^13^ In order for substrates to access these active sites for catalysis, the surface ligands must typically be removed. One common approach for removing surface ligands on nanoparticles is through high-temperature reductive annealing. For example, in our previous thermocatalytic CO_2_ hydrogenation reactions with α-MoC_1–*x*_ nanoparticles, the catalysts were activated at 450 °C under a reducing atmosphere (95% H_2_/5% Ar flow) for 2 h to remove the oleylamine ligands; this activation temperature is significantly higher than the synthesis temperature of the α-MoC_1–*x*_ nanoparticle catalysts (290–320 °C) and the CO_2_ hydrogenation temperature (300 °C).^5,12^ Aside from being energy-intensive, this method may lead to particle sintering or coking of the surface ligands, which blocks surface active sites and decreases catalytic activity.^15,16^ Treating the nanoparticles with acid is another common approach for stripping away the surface ligands, although this method is insufficient if the ligands are tightly bound to the surface and, in some cases, can poison or etch the nanoparticle catalysts.^17^

We propose an alternative method to remove the native, long-chain aliphatic ligands, which is to perform a postsynthetic exchange for new ligands that are thermally labile yet still impart solution processability. Once deposited on a surface, the thermally labile ligands can be easily removed by mild heating, circumventing the need for high-temperature thermolysis. This concept has been previously demonstrated for colloidal quantum dots; upon solution deposition of neat quantum dot thin films, a mild heat treatment improves quantum dot spatial and electronic coupling and increases carrier mobility without particle sintering through the expulsion of the thermally labile ligands.^18−21^ However, thermally labile ligands have not yet been applied to nanoparticle catalysts as a way to remove ligands and reveal actives sites during catalyst activation. While short-chain ligands have been shown to successfully exchange with native ligands to reduce the overall carbon content on the surface of metal nanoparticle catalysts,^22,23^ the idea of implementing thermally labile ligands for facile thermal decomposition during catalyst activation has not been explored.

Herein, we report the first successful exchange of native oleylamine ligands for shorter chain ligands on the surface of α-MoC_1–*x*_ nanoparticle catalysts. We use solution ^1^H NMR spectroscopy as a probe to investigate ligand exchange and then further extend this concept to use a thermally labile ligand, *tert*-butylamine (*t*-BuNH_2_). Characterization of the *t*-BuNH_2_ exchanged nanoparticles using thermogravimetric analysis–mass spectrometry (TGA-MS) and FT-IR spectroscopy confirms the effectiveness of this exchange method. Employing CO chemisorption reveals that after a mild thermal treatment at 250 °C the *t*-BuNH_2_–MoC_1–*x*_ catalyst activates ca. 25% of the total binding sites compared to none for the MoC_1–*x*_ catalyst with native oleylamine ligands. CO_2_ hydrogenation catalysis also demonstrates that the ligand exchanged material and the as-prepared nanoparticle catalysts have distinctly different surfaces because of their different ligand environments.

## Experimental Section

### α-MoC_1–*x*_ Nanoparticle Synthesis

Oleylamine (70% technical grade) was purchased from Sigma-Aldrich and dried by heating to 120 °C under vacuum for ca. 5 h prior to use. Mo(CO)_6_ (98%) was purchased from Sigma-Aldrich and used as received. In a standard procedure, Mo(CO)_6_ (264 mg, 1.00 mmol) was added to a three-neck round-bottom flask fitted with a reflux condenser and two septa and then briefly evacuated and filled with N_2_ three times using standard Schlenk techniques. Oleylamine (12 mL, 36 mmol) was injected under flowing N_2_ and heated rapidly in a thermostat-controlled sand bath to 320 °C and then maintained this temperature for 1 h. The reaction mixture was then allowed to cool to ambient temperature naturally. Approximately 4 mL of hexanes was used to assist in the transfer of the cooled reaction mixture, which was then split equally between two 50 mL centrifuge tubes. The centrifuge tubes were then briefly bath sonicated and vortexed mixed. The product was precipitated by the addition of 32 mL of acetone to each centrifuge tube followed by centrifugation (6000 rpm, 20 min). The clear supernatant was decanted and discarded, and the black nanoparticle pellet was redispersed in 0.5 mL of CHCl_3_. After vortex mixing and bath sonicating the suspension, the nanoparticles were reprecipitated using 39 mL of ethanol followed by centrifugation (6000 rpm, 10 min). This washing step with CHCl_3_ and ethanol was then performed once more. The resulting nanoparticle pellet was redispersed in CHCl_3_ and dried overnight under flowing N_2_.

### ^1^H NMR Titrations with Undec-10-en-1-amine (UDAm)

Undec-10-en-1-amine (95%) and ferrocene (98%) were purchased from Sigma-Aldrich and used as received. Toluene-*d*_8_ (99+%) was purchased from Acros Organics and used as received. A 1 mL suspension of 8 mg of the colloidal MoC_1–*x*_ nanoparticles in CHCl_3_ was transferred to a J. Young NMR tube and dried overnight under vacuum. The nanoparticles were then redispersed in 0.8 mL of toluene-*d*_8_ in the J. Young tube by bath sonication for 5 min. Additionally, 5 μL of a 1 mM solution of ferrocene in toluene-*d*_8_ was added to the J. Young tube as an internal standard. Titrations were performed with 10 μL aliquots of a 0.23 M solution of UDAm in 250 μL of toluene-*d*_8_.

### Ligand Exchange with *tert*-Butylamine (*t*-BuNH_2_)

*tert*-Butylamine (98%) was purchased from Sigma-Aldrich and used as received. In a standard procedure, 32 mg of MoC_1–*x*_ was dried and redispersed in 1 mL of toluene in a three-neck round-bottom flask fitted with a condenser and two septa. Excess *t*-BuNH_2_ (19.6 mmol) was then added to the flask. The flask was kept under flowing nitrogen and heated to 40 °C in a sand bath for 1 h. The suspension was then transferred to a 50 mL centrifuge tube, and 18 mL of ethanol was added to precipitate the product for isolation. Attempts to achieve higher degrees of ligand exchange resulted in a decrease in isolated particle yield due to extensive washing.

### Acid Treatment with Trifluoroacetic Acid (TFA)

Trifluoroacetic acid (99%) was purchased from Sigma-Aldrich. In a typical experiment, 500 μL of TFA was added to a suspension of 50 mg of MoC_1–*x*_ nanoparticles in toluene (2 mL). The suspension was allowed to stir at room temperature overnight and then transferred to a 50 mL centrifuge tube and washed once with ethanol. TGA was performed to obtain the resulting organic content after acid treatment.

### Catalyst Support

Following centrifugation, the isolated α-MoC_1–*x*_ nanoparticles were redispersed in ca. 5 mL of CHCl_3_ and slowly added to a rapidly stirring suspension of 1 g of Vulcan XC 72 R carbon dispersed in ca. 60 mL of CHCl_3_. The solution was bath sonicated for 5 min and rapidly stirred overnight. The catalyst was separated via centrifugation (6000 rpm, 10 min) and dried under vacuum at room temperature.

### Solution ^1^H NMR Spectroscopy

All solution ^1^H NMR spectra were collected on a Varian 600 MHz VNMRS spectrometer with 16 scans, a 30 s relaxation delay, a 45° pulse angle, and an acquisition time of 2.726 s. The ^1^H NMR spectra were normalized to the ferrocene peak at δ 4.16 ppm. Toluene-*d*_8_ was used as the deuterated solvent.

### Powder X-ray Diffraction (XRD)

Powder XRD patterns were collected on a Rigaku Ultima IV diffractometer operating at 40 mA and 44 kV with a Cu Kα X-ray source (λ = 1.5406 Å).

### Transmission Electron Microscopy (TEM)

TEM images were acquired with a JEOL JEM2100F (JEOL Ltd.) microscope operating at 200 kV. Each sample was prepared by drop-casting on 400 mesh Cu grids coated with a lacey carbon film (Ted Pella, Inc.) and dried overnight under vacuum at room temperature.

### Thermogravimetric Analysis (TGA)

Thermogravimetric analysis of the MoC_1–*x*_ nanoparticles was performed on a TGA Q50 instrument. To determine the organic ligand content, ca. 5 mg of the nanoparticles was isolated (after work-up and drying) and dried at 40 °C for 2 h before being heated to 450 °C under flowing N_2_ at a heating rate of 10 °C min^–1^.

### Thermogravimetric Analysis–Mass Spectrometry (TGA-MS)

TGA-MS analysis was performed using a Hiden Analytical HPR-20 EGA benchtop gas analysis system (equipped with a 20 mL min^–1^ capillary) attached to a Netzsch STA 449 F3 Jupiter. To determine the molecular weight of the decomposition products, ca. 10 mg of sample was used and heated to 450 °C at a rate of 10 °C min^–1^ and analyzed in the range of *m*/*z* = 14–200.

### FT-IR Spectroscopy

FT-IR spectra were acquired on a Bruker Vertex 80 spectrophotometer using 16 scans, 4 cm^–1^ resolution, 4000–400 cm^–1^ spectral range, and absorbance units as the operational parameters. An internal standard (Fe_4_[Fe(CN)_6_]_3_) was used to obtain semiquantitative results. KBr (4.19 g, analytical reagent grade, dried for 5 days at 180 °C) was finely powdered with an alumina mortar and pestle before the addition of Fe_4_[Fe(CN)_6_]_3_ (5.6 mg), which was finely mixed together (total griding time ca. 30 min). Dried MoC_1–*x*_ samples (4.6 mg) were then added to a 200.0 mg preground portion of the KBr/Fe_4_[Fe(CN)_6_]_3_ mixture and finely ground. A portion of this sample (∼20 mg) was pressed into a thin disc using a 9 mm diameter hand-operated screw press and then immediately introduced into the spectrometer. A background spectrum was performed with a similarly prepared KBr pellet. The spectra were normalized to the absorbance at 2088.53 cm^–1^, which is judged to be the center of the main ν(C≡N) stretch in the internal standard.

### Chemisorption

Catalytic site densities of the carbon-supported α-MoC_1–*x*_ nanoparticles were determined by carbon monoxide (CO) chemisorption at 50 °C and hydrogen (H_2_) chemisorption at 250 °C over the pressure range of 200–500 Torr using a Quantachrome Autosorb 1-C gas sorption instrument. Analyses at different temperatures for each titrant molecule follows literature precedent for activated processes (i.e., higher temperatures needed for H–H bond cleavage to form H* on carbides) versus nonactivated processes (i.e., adsorption of CO at low temperature to prevent C–O bond cleavage at higher temperatures).^24,25^ Samples (ca. 175 mg) were reduced with flowing UHP H_2_ at 250 or 450 °C for 2 h, followed by evacuation at the reduction temperature for 8 h. The site density (units of μmol_CO*_/g_cat_ or μmol_H*_/g_cat_) was determined from the difference of the combined and weak isotherms extrapolated to zero pressure with a zero slope.

### Catalytic Evaluation

The carbon-supported α-MoC_1–*x*_ nanoparticle catalysts were evaluated for their performance in the CO_2_ hydrogenation reaction following similar conditions to those previously described.^5,12^ Catalyst (ca. 0.4–0.7 g) was loaded in a 1/4 in. inner diameter stainless steel tubular reactor and pretreated under 95% H_2_/5% Ar flow (50 sccm) at 250 °C for 2 h with a 2 °C min^–1^ ramp rate. After reduction, the temperature was adjusted to the desired reaction temperature of 250 °C. Gas flow rates for CO_2_ and 95% H_2_/5% Ar were adjusted to achieve the same weight-hourly space velocity (WHSV) of ca. 40 h^–1^ based on the Mo content of the catalyst measured by ICP-OES. A feed gas composition of 26:70:4 mol % for CO_2_:H_2_:Ar, respectively (corresponding to a molar H_2_:CO_2_ ratio in the feed of 2.7), was employed. Product analysis was performed online by an Agilent Technologies 7890B gas chromatograph equipped with flame ionization detectors (FIDs) and thermal conductivity detectors (TCDs). Conversion was calculated as ∑(molar flow rate of C in all products)/(molar flow rate of inlet CO_2_). The C-selectivity of product *i* was calculated as (molar flow rate of C in product (*i*)/∑(molar flow rate of C in all products).

## Results and Discussion

### As-Prepared MoC_1–*x*_ Nanoparticles

**Figure 1:**
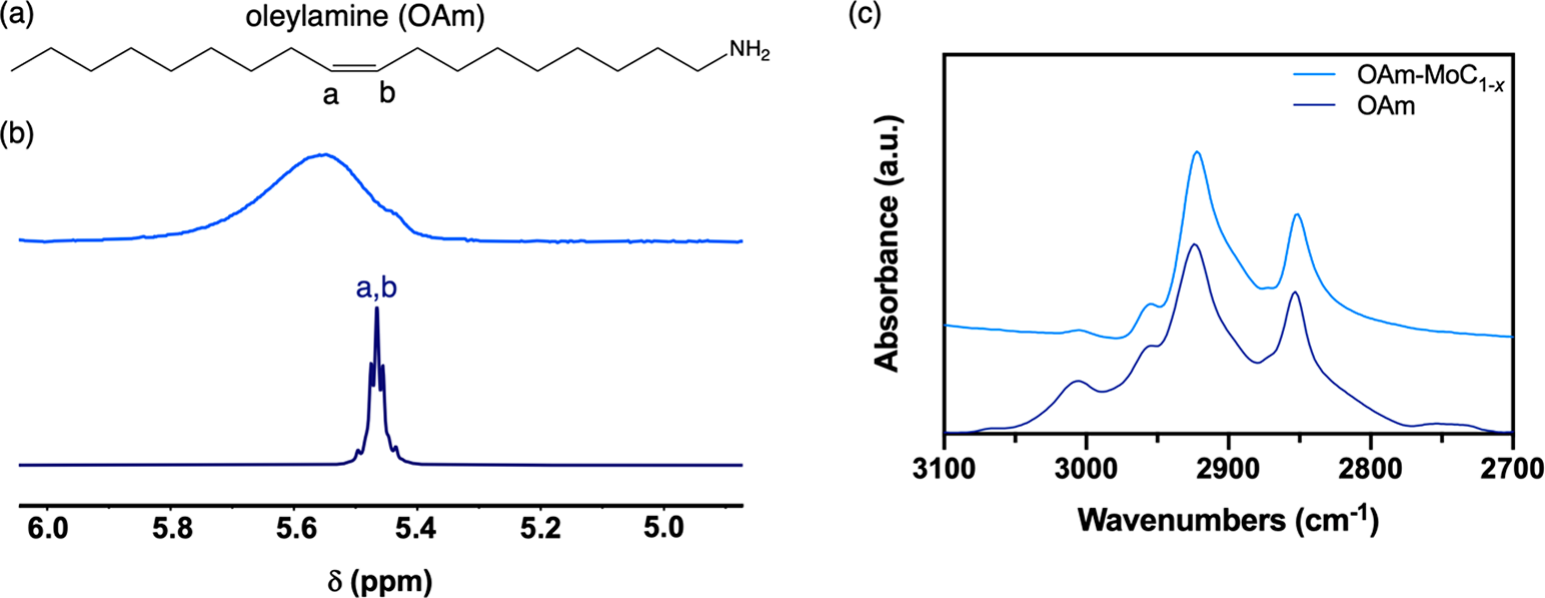
Surface ligand characterization of as-prepared OAm-MoC_1–*x*_ nanoparticles. (a) Structure of oleylamine and (b) solution ^1^H NMR spectra of the OAm-MoC_1–*x*_ nanoparticles (top) and free oleylamine (bottom) in toluene-*d*_8_. (c) ν(C–H) stretching region of the FT-IR spectra of the OAm-MoC_1–*x*_ nanoparticles (top) and free oleylamine (bottom).

The colloidal MoC_1–*x*_ nanoparticle catalysts were prepared through a solution-phase thermolytic decomposition of Mo(CO)_6_ in neat oleylamine (OAm), adapted from a previously reported method.^5^ This synthesis yields small multipodal nanoparticles ca. 2 nm in diameter that crystallize in the FCC α-phase of molybdenum carbide (*vide infra*). The ligands on the surface of these nanoparticles were characterized though solution ^1^H NMR and FT-IR spectroscopies. Solution ^1^H NMR is a powerful tool for characterizing the ligands on nanoparticle surfaces because of distinct changes in both line widths and chemical shifts that are observed with ligands bound to the surface or free in solution.^26^ The solution ^1^H NMR spectrum of the MoC_1–*x*_ nanoparticles capped with oleylamine (OAm-MoC_1–*x*_) displays a diagnostic feature for the alkenyl protons (Figure 1a) of bound oleylamine. In toluene-*d*_8_, these alkenyl protons of oleylamine shift downfield from δ 5.47 ppm for free ligand to δ 5.59 ppm when bound to the surface (Figure 1b). The clear broadening and downfield shift of the alkenyl peaks confirm that oleylamine is bound to the surface of the nanoparticles. It should be noted that the alkenyl region is used to probe ligand binding because the upfield region of the ^1^H NMR spectrum is typically complicated by overlapping resonances from the aliphatic protons in these long-chain ligands.^27^ Additionally, the ν(C–H) stretching envelope of the FT-IR spectrum of OAm-MoC_1–*x*_ agrees with that of oleylamine, with stretching bands at 3000, 2950, 2920, and 2850 cm^–1^ (Figure 1c). The clear ν(C–H) stretching bands in range 3000–2850 cm^–1^, along with the shift of these bands to lower wavenumbers compared to free oleylamine, also confirm the ligand binding.^28−30^ The native oleylamine surface ligand density on the as-prepared MoC_1–*x*_ nanoparticles is ca. 3 nm^–2^, as determined by thermogravimetric analysis (TGA).^26^ This calculated surface density agrees well with that of a theoretical monolayer of 2.8 oleylamine nm^–2^ (using a ligand footprint of 0.36 nm^2^),^31−33^ assuming particle sphericity.

### Ligand Exchange and Characterization

The first attempt to remove native oleylamine ligands on the MoC_1–*x*_ nanoparticle surface involved a treatment with trifluoroacetic acid (TFA), which has successfully removed long-chain insulating ligands on the surface of colloidal nanocrystals and is a common technique for ligand removal in general, as aforementioned.^34−36^ However, after the addition of 500 μL of TFA (i.e., 50× molar excess compared to previous reports) to a colloidal suspension of MoC_1–*x*_ nanoparticles and allowing the suspension to stir rapidly overnight, no nanoparticles precipitated out of solution, which would be expected for effective ligand stripping. After isolating the TFA-treated MoC_1–*x*_ nanoparticles, TGA and FT-IR spectroscopy (Figures S1 and S2, respectively) were performed and compared to the as-prepared OAm-MoC_1–*x*_ nanoparticles. The resulting TGA traces have the same mass loss profile, indicating no removal of native oleylamine ligands with TFA treatment. Additionally, the FT-IR traces of the two samples are almost identical, further demonstrating that this acid treatment is ineffective in removing the native oleylamine ligands.

These results necessitated a new approach. To enable the mild thermolytic removal of the stabilizing ligands on the MoC_1–*x*_ nanoparticle surfaces, we looked to replace the native oleylamine ligands with a much smaller C-4 *tert*-butylamine (*t*-BuNH_2_) ligand. An amine ligand was chosen because we know that the native oleylamine ligands can be removed (albeit at high temperatures) without any of the ligand decomposition products poisoning the resulting MoC_1–*x*_ nanoparticle catalyst for CO_2_ hydrogenation. *tert*-Butylamine is commercially available and inexpensive and, because of its steric bulk, should not pack tightly on the nanocrystal surface,^37^ thereby minimizing the overall organic ligand content. Moreover, the presence of a tertiary carbon center in *t*-BuNH_2_ should enable the low-temperature decomposition of this ligand (*vide infra*).

**Figure 2:**
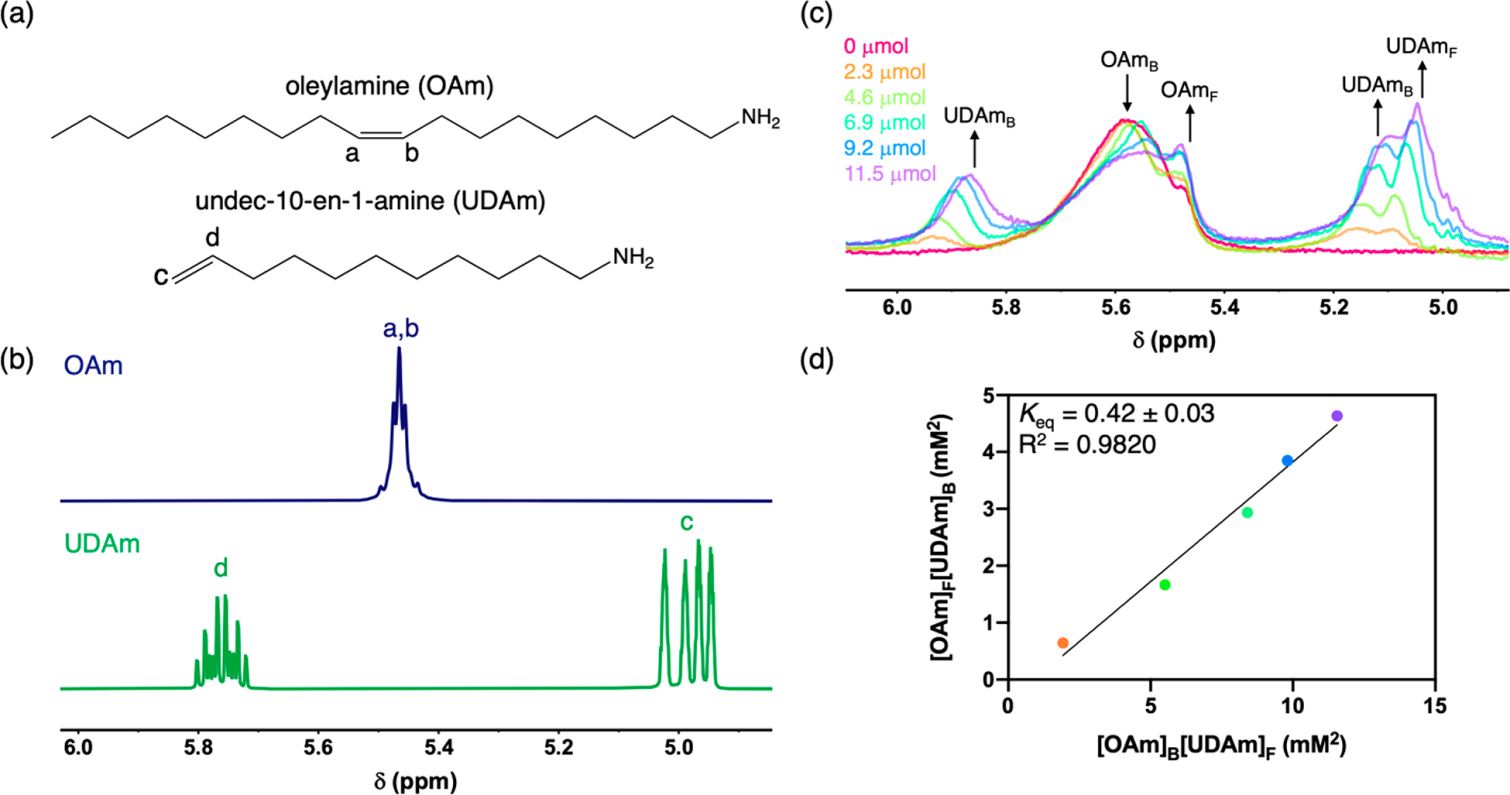
(a) Structures of oleylamine and undec-10-en-1-amine with labels corresponding to (b) solution ^1^H NMR spectra of the free ligands in toluene-*d*_8_. (c) Room-temperature solution ^1^H NMR spectra of a 0.1 M OAm-MoC_1–*x*_ nanoparticle suspension in toluene-*d*_8_ titrated with increasing amounts (0–11.5 μmol) of UDAm, showing both free (F) and bound (B) fractions of each ligand. (d) Plot of [OAm]_F_[UDAm]_B_ vs [OAm]_B_[UDAm]_F_. The slope of the resulting line returns a *K*_eq_ value for the ligand exchange between OAm and UDAm.

*tert*-Butylamine does not have distinct chemical shifts from oleylamine that would allow for the ligand exchange reaction to be followed by solution ^1^H NMR. Therefore, to evaluate the feasibility of exchanging a shorter-chain primary amine ligand for oleylamine on the surface of colloidal MoC_1–*x*_ nanoparticles, we employed undec-10-en-1-amine (UDAm) as a probe molecule for ligand exchange. UDAm has distinct vinylic protons at ca. δ 5.76 and 4.98 ppm compared to the internal alkenyl protons of oleylamine (Figure 2a) that resonate at δ 5.47 ppm (Figure 2b), making it ideal to simultaneously track the free and bound fractions of each ligand and allow the surface equilibrium to be calculated.^27,32,38^ While UDAm has a different steric profile than *t*-BuNH_2_, it allows the feasibility of the ligand exchange of oleylamine for another primary amine to be easily gauged by solution NMR spectroscopy.

Ligand exchange reactions were performed with 8 mg of purified OAm-MoC_1–*x*_ nanoparticles in a colloidal suspension in toluene-*d*_8_. The total amount of oleylamine in this sample was quantified by integrating the alkenyl resonances against an internal ferrocene standard. The ligand exchange was then performed by titrating an equimolar amount of UDAm into the suspension of OAm-MoC_1–*x*_ nanoparticles at room temperature. Upon the addition of UDAm, there is a decrease in bound oleylamine (δ 5.59 ppm) with a concomitant increase in the upfield resonance for free oleylamine (δ 5.47 ppm). In addition, with increasing amounts of UDAm, there is a clear broadening and downfield shift of the vinylic resonances from UDAm (δ 5.93 and 5.15 ppm) compared to free UDAm (δ 5.76 and 4.98 ppm), indicating that UDAm is binding to the surface of the nanoparticles and displacing native oleylamine in the process (Figure 2c). After addition of 0.5 mol equiv of UDAm relative to the starting oleylamine, ca. 25% of the bound oleylamine is displaced. Plotting [OAm]_F_[UDAm]_B_ versus [OAm]_B_[UDAm]_F_ over the titration series returns a straight line with a slope that gives an equilibrium constant *K*_eq_ = 0.42 ± 0.03 (Figure 2d). This equilibrium constant agrees with the average equilibrium constant calculated by quantifying the bound and free fractions of oleylamine and UDAm over the same titration series (Table S1). The small *K*_eq_ for this reaction suggests that there is not a large driving force at room temperature, as might be expected for a ligand exchange of one primary amine for another. To try and push the exchange further, we performed a forced ligand exchange reaction with a stoichiometric excess of UDAm (2.8 equiv relative to bound oleylamine) with mild heating at 40 °C for 1 h. After forced ligand exchange, ca. 50% of the bound oleylamine is displaced, as assessed by ^1^H NMR spectroscopy, which is an improvement on the stoichiometric, room temperature exchange (Figure S4).

**Figure 3:**
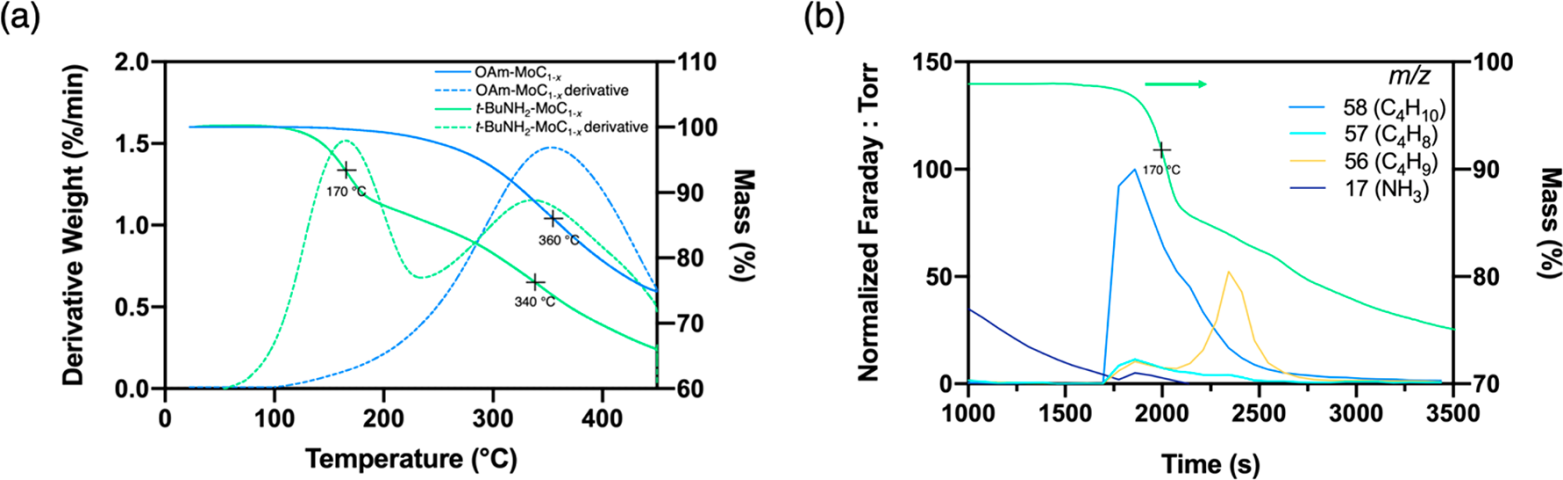
(a) TGA traces (solid lines) of the OAm-MoC_1–*x*_ nanoparticles and the exchanged *t*-BuNH_2_-MoC_1–*x*_ nanoparticles along with their respective derivative curves (dotted lines). (b) TGA-MS of the *t*-BuNH_2_-MoC_1–*x*_ nanoparticles, revealing the major decomposition products at the low-temperature mass loss event.

Given the success of the forced ligand exchange reaction with UDAm, we applied these same conditions for the installation of *t*-BuNH_2_ onto the OAm-MoC_1–*x*_ nanoparticle surface. In a typical reaction, 30 mg of OAm-MoC_1–*x*_ nanoparticles was added to an excess of *t*-BuNH_2_ and heated to 40 °C for 1 h. The resulting ligand exchange product was purified once by precipitation with ethanol and then resuspended in a nonpolar solvent, such as toluene or hexanes. The ligand exchanged MoC_1–*x*_ remained colloidally stable after purification, without the need for an additional donor solvent for stabilization.^21^ TGA of the *t*-BuNH_2_-MoC_1–*x*_ exchanged nanoparticles was compared to the as-prepared OAm-MoC_1–*x*_ nanoparticles (ambient to 450 °C, 10 °C min^–1^), as shown in Figure 3a. Both samples were thoroughly dried at 40 °C (boiling point of *t*-BuNH_2_ = 46 °C) for 2 h before starting each run. The TGA trace for the *t*-BuNH_2_-MoC_1–*x*_ exchanged nanoparticles shows two distinct mass loss events. The first mass loss event is at ca. 170 °C, which is not seen in the native OAm-MoC_1–*x*_ nanoparticles. A second mass loss event occurs at ca. 340 °C and is attributed to loss/decomposition of oleylamine as it is the only mass loss event observed in the as-prepared OAm-MoC_1–*x*_ nanoparticles. Additionally, the derivative of each TGA trace is plotted, which highlights inflection points in the curve, further illustrating that the *t*-BuNH_2_-MoC_1–*x*_ exchanged nanoparticles have two distinct mass loss events, while the as-prepared OAm-MoC_1–*x*_ nanoparticles have only one. Therefore, from TGA analysis, the exchanged nanoparticles possess ca. 7.8 mmol of *t*-BuNH_2_ mg^–1^ nanoparticles and ca. 5.6 mmol of oleylamine mg^–1^ nanoparticles. This corresponds to ca. 60% of the surface ligands being *t*-BuNH_2_, which is similar to the degree of ligand exchange achieved with excess UDAm quantified by ^1^H NMR (*vide supra*). TGA–mass spectrometry (TGA-MS) was used to confirm the identity of the *t*-BuNH_2_ ligand corresponding to the low-temperature mass loss event in the exchanged nanoparticles. During the ca. 170 °C mass loss event, C_4_H_10_, C_4_H_8_, C_4_H_9_ (assigned to isobutane, isobutene, and *t*-Bu^+^), and NH_3_ were observed as the major volatile decomposition products, as displayed in Figure 3b. These products are accounted for through facile homolytic bond cleavage and H-atom transfer steps. Similar decomposition and fragmentation products have been reported for the *tert*-butylthiol ligand analogue.^21^ In comparison, TGA-MS of the OAm-MoC_1–*x*_ nanoparticles in this same temperature range gave no detectable volatiles by mass spectrometry.

**Figure 4:**
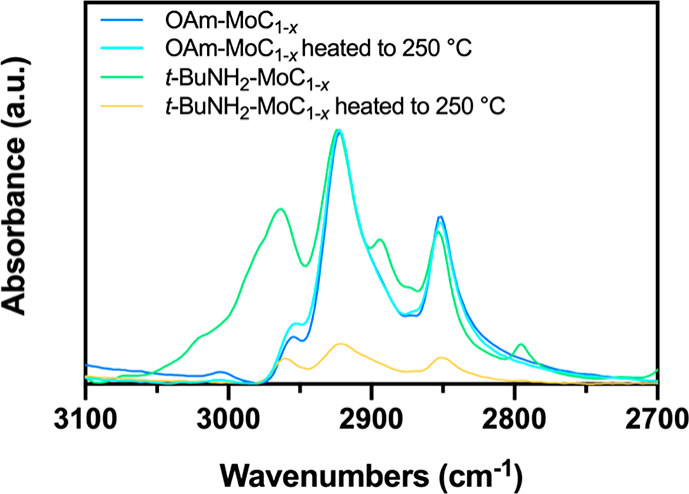
ν(C–H) stretching region of the FT-IR spectra of MoC_1–*x*_ nanoparticles (KBr matrix). Thermally treated OAm-MoC_1–*x*_ and *t*-BuNH_2_-MoC_1–*x*_ exchanged nanoparticles were heated to 250 °C. The spectra were normalized to the 2089 cm^–1^ ν(C≡N) stretching band of a Fe_4_[Fe(CN)_6_]_3_ internal standard (not shown).

Decomposition of the *t*-BuNH_2_ ligand on the exchanged MoC_1–*x*_ nanoparticles was further corroborated by semiquantitative FT-IR spectroscopy. The ν(C–H) stretching region of the FT-IR spectra of OAm-MoC_1–*x*_, the *t*-BuNH_2_–MoC_1–*x*_ exchanged nanoparticles, and each respective sample after being heated to 250 °C is provided in Figure 4. A thermal treatment of 250 °C was chosen because it is past the end of the first decomposition event in the TGA of the *t*-BuNH_2_-MoC_1–*x*_ nanoparticles. The FT-IR spectrum of the OAm-MoC_1–*x*_ nanoparticles has distinct ν(C–H) stretches at 3000, 2950, 2920, and 2850 cm^–1^ which indicate oleylamine is present on the surface, as aforementioned. After heating the OAm-MoC_1–*x*_ nanoparticles to 250 °C, there is no significant change in ν(C–H) stretching intensity observed in the FT-IR spectrum, indicating that no oleylamine has left the surface. While the FT-IR spectrum of the *t*-BuNH_2_-MoC_1–*x*_ exchanged nanoparticles has similar ν(C–H) stretches to the OAm-MoC_1–*x*_ nanoparticles, there are also unique stretching bands present at 3015, 2960, 2890, and 2790 cm^–1^ which correspond to bound *t*-BuNH_2_. Upon mild heating of the *t*-BuNH_2_-MoC_1–*x*_ exchanged nanoparticles to 250 °C, there is a drastic decrease in the overall organic content, as evidenced by a reduction in the ν(C–H) stretching intensity, and the resulting spectrum only contains the characteristic ν(C–H) stretches of oleylamine. This suggests that after a mild heating treatment at 250 °C, *t*-BuNH_2_ is removed from the surface. Additionally, after heating both samples to 450 °C, all characteristic ν(C–H) stretches in this region disappear, indicating that the organic ligands on the surface are completely removed at this higher temperature (Figure S3).

**Figure 5:**
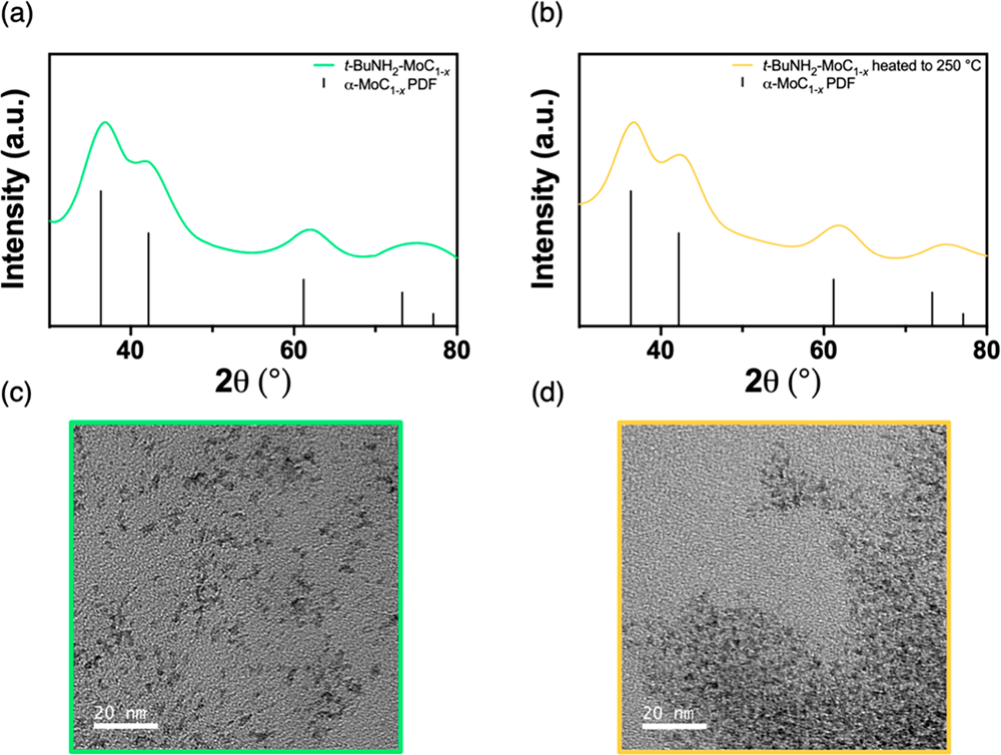
(a, b) XRD patterns and (c, d) TEM images of unsupported *t*-BuNH_2_-MoC_1–*x*_ nanoparticles before and after heating to 250 °C, respectively.

The powder X-ray diffraction (XRD) and selected area electron diffraction (SAED) patterns of the *t*-BuNH_2_-MoC_1–*x*_ exchanged nanoparticles can be indexed to the FCC α-phase of molybdenum carbide and display significant peak broadening, consistent with the as-prepared OAm-MoC_1–*x*_ nanoparticles (Figures 5a, S5a, and S7).^5,12^ Scherrer analysis of the XRD patterns returns a crystallite size 2.0 nm before and after ligand exchange, indicating that the ligand exchange process does not affect the nanoparticle size or crystallinity. XRD and SAED patterns of both the OAm-MoC_1–*x*_ and *t*-BuNH_2_-MoC_1–*x*_ exchanged nanoparticles heated to 250 °C also show no change, proving the absence of a phase transition and any significant particle sintering (Figures 5b, S5b, and S7). The size derived from Scherrer broadening is qualitatively similar to the size of the nanoparticles determined by transmission electron microscopy (TEM). The lattice fringes of the *t*-BuNH_2_-MoC_1–*x*_ and OAm-MoC_1–*x*_ nanoparticles, and each respective material heated to 250 °C, was observed through high-resolution TEM and confirms single crystalline particles (Figure S6). The measured *d*-spacing (0.25 nm) corresponds to the (111) plane and agrees with previous reports.^5,12^ While the nanoparticle size does not change upon heating, it is important to note that the unsupported *t*-BuNH_2_-MoC_1–*x*_ exchanged nanoparticles heated to 250 °C do show a significant decrease in interparticle separation (Figures 5c,d), consistent with agglomeration, but not sintering. This is to be expected since ca. 60% of the total ligands are being removed during this heating step with the ligand exchanged nanoparticles.

**Figure 6:**
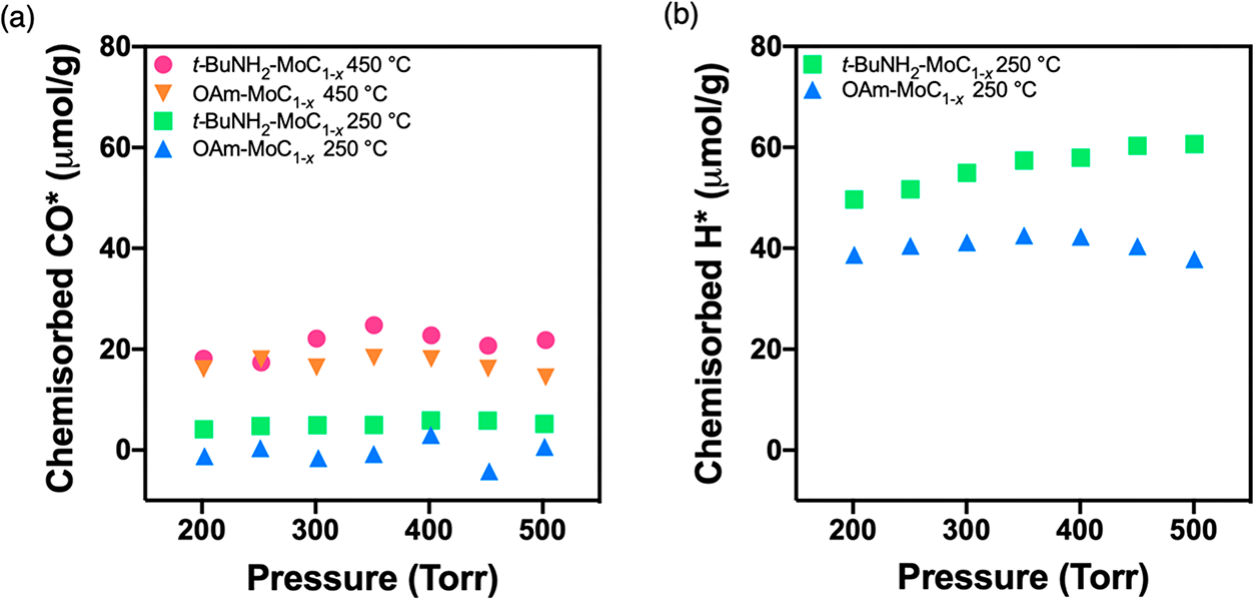
Plot of mass-normalized strong (a) CO and (b) H_2_ chemisorption as a function of pressure for OAm-MoC_1–*x*_/C and the *t*-BuNH_2_-MoC_1–*x*_/C.

Carbon monoxide (CO) and hydrogen (H_2_) chemisorption was used to compare the effect of ligand exchange and low-temperature thermal activation on surface site availability for carbon-supported OAm-MoC_1–*x*_ and *t*-BuNH_2_-MoC_1–*x*_ nanoparticles. Like our previous reports, the respective nanoparticles were immobilized on Vulcan XC72R carbon, yielding materials having 3.7 and 6.1 wt % Mo for OAm-MoC_1–*x*_/C and *t*-BuNH_2_-MoC_1–*x*_/C, respectively.^5,12^ Both the OAm-MoC_1–*x*_ and *t*-BuNH_2_-MoC_1–*x*_ nanoparticle catalysts were able to be supported identically, demonstrating the solution processability of the ligand exchanged MoC_1–*x*_ nanoparticles. The *t*-BuNH_2_ exchanged nanoparticles exhibited a CO* site density of 5.10 μmol_CO_/g_cat_ after the 250 °C treatment, whereas the as-prepared OAm-MoC_1–*x*_/C had no CO uptake (Figure 6a). This difference in CO* site density after the 250 °C pretreatment is in accord with the TGA and FT-IR data, indicating that this pretreatment temperature was not sufficient to generate surface binding sites for CO from the oleylamine-terminated nanoparticles but could activate ca. 25% of the total binding sites for the *t*-BuNH_2_ exchanged nanoparticles (comparing to activation at 450 °C). After the 250 °C reductive treatment, both catalysts activated H_2_ and formed strongly bound H* (Figure 6b). Similar to the CO* site density results, the *t*-BuNH_2_ exchanged nanoparticles exhibited a greater H* site density, giving a nearly 40% increase to 56.1 μmol_H*_/g_cat_ versus 40.5 μmol_H*_/g_cat_ for the parent material.

**Figure 7:**
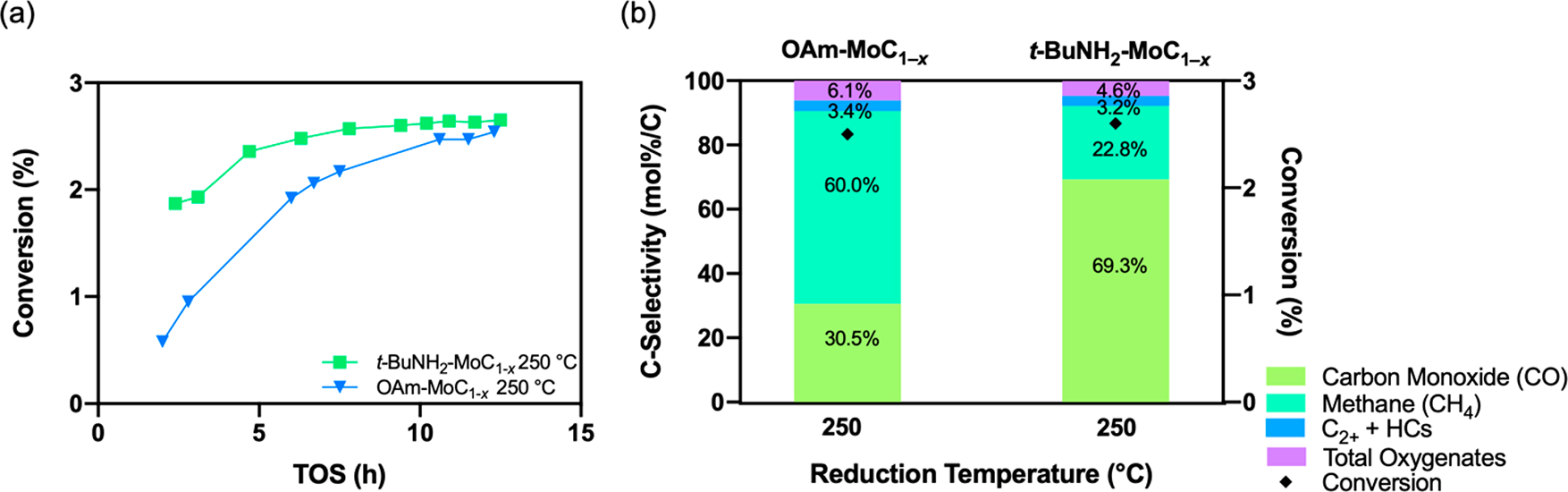
(a) Conversion vs time on stream (TOS) during CO_2_ hydrogenation catalysis evaluation and (b) conversion and product selectivities at ca. 10 h TOS for the carbon-supported MoC_1–*x*_ catalysts reduced at 250 °C.

The effects of ligand exchange and reduction temperature (i.e., ligand removal at low temperature) on the catalytic activity of the as-prepared OAm-MoC_1–*x*_ and *t*-BuNH_2_–MoC_1–*x*_ exchanged nanoparticles supported on carbon were further evaluated in the CO_2_ hydrogenation reaction at 250 °C and 2 MPa. As illustrated in Figure 7a, an induction period was observed where the conversion increased over the first 10 h time on stream (TOS). This behavior is similar to our previous reports of the performance of carbon-supported MoC_1–*x*_ nanoparticle catalysts.^5,12^ The OAm-MoC_1–*x*_/C catalyst had a very low initial conversion (0.6% at 2.0 h TOS), suggesting insufficient active site availability of the MoC_1–*x*_ nanoparticles that retained the high ligand coverage and steric bulk of the oleylamine ligand after the low-temperature activation, in accord with the lack of CO chemisorption (Table S2 and Figure 6a). During the 12 h reaction period, the high H_2_ pressure appeared to facilitate catalyst activation, gradually leading to increased conversion during the induction period and reaching 2.5% at 10.6 h TOS. On the other hand, greater initial activity was observed for the ligand exchanged *t*-BuNH_2_-MoC_1–*x*_/C catalyst (1.9% at 2.4 h TOS) indicative of more efficient catalyst activation during the mild pretreatment step. Considering the TGA-MS, FT-IR, and CO chemisorption data presented, this is attributed to the more facile thermal decomposition of the *t*-BuNH_2_ ligand than oleylamine. An increase in conversion was still observed during the induction period over *t*-BuNH_2_-MoC_1–*x*_/C, but the change was rather modest (i.e., from 1.9% at 2.4 h to 2.6% at ca. 10 h TOS) compared to that observed over OAm-MoC_1–*x*_/C (0.6% at 2.0 h to 2.5% at ca. 10 h TOS).

Product selectivity was compared after the induction period (ca. 10 h TOS) for both catalysts activated at 250 °C (Figure 7b). The dominant products were CO and methane with minor products of methanol and dimethyl ether (represented as “total oxygenates”) and C_2+_ hydrocarbons (“C_2+_ HCs”). Interestingly, greater selectivity to methane, and correspondingly lower selectivity to CO, was observed for the OAm-MoC_1–*x*_/C catalyst reduced at 250 °C (60.0% CH_4_ and 30.5% CO) compared to the ligand exchanged *t*-BuNH_2_-MoC_1–*x*_/C catalyst (22.8% CH_4_ and 69.3% CO). This major difference in selectivity suggests a significant catalytic consequence of the remaining oleylamine ligands after the mild 250 °C pretreatment. One possibility is incomplete reduction of the carbide surface due to ligand retention, which is supported by the low initial activity and long induction period described above. Additionally, the lack of CO* site density but moderate H* site density suggests that another possibility for the high methane selectivity for the OAm-MoC_1–*x*_/C catalyst is a comparatively high H*/CO_x_* ratio on this catalyst surface after the induction period, leading to complete hydrogenation to methane.^39−41^ In contrast, the *t*-BuNH_2_–MoC_1–*x*_/C catalyst reduced at 250 °C exhibited greater CO* site density with a H* site density in the same order-of-magnitude, suggesting a lower H*/CO_x_* ratio, and subsequently, a lower hydrogenation selectivity to CH_4_. Comparing the selectivity for the *t*-BuNH_2_-MoC_1–*x*_/C catalyst reduced at 250 °C to both catalysts pretreated at 450 °C revealed similar product slates (Figure S8). This observation suggests that the mild pretreatment of the ligand exchanged *t*-BuNH_2_-MoC_1–*x*_/C catalyst generated CO_*x*_ active sites similar to those generated after a high-temperature reduction for both catalysts, and further, this data highlights the inability of generating these sites for the as-prepared, oleylamine-terminated catalyst at the lower reduction temperature. Lastly, the selectivity away from the terminal product methane and toward CO, which can subsequently lead to the desired C_2+_ products, represents an advantageous shift in the catalytic selectivity enabled at lower temperature by the ligand exchange process.^5,42,43^

## Conclusions

We established a new method of activating nanoparticle catalysts under mild conditions via exchange with thermally labile ligands. Ligand exchange with small thermally labile ligands had been previously applied to colloidal quantum dots to retain their solution processability in organic solvents but vastly improves inter-nanocrystal spatial and electronic coupling upon thin film deposition and mild heating.^18−21^ This was used with great success to increase the efficiency of quantum dot-based solar cells,^44,45^ enable direct optical lithography,^46^ and immobilize 2D crystalline quantum dot superlattices.^47^ In each of these applications, the thermally labile ligands allow for ligand removal at sufficiently low temperatures that do not induce quantum dot sintering or loss of quantum confinement. Here, we apply the same concept toward a different end by installing a thermally labile ligand on MoC_1–*x*_ nanoparticle catalysts. The *t*-BuNH_2_ ligand provides excellent colloidal dispersibility in organic solvents, which allows the MoC_1–*x*_ nanoparticles to be supported on carbon using standard methods. Once supported, the ligand exchanged *t*-BuNH_2_-MoC_1–*x*_/C catalyst can be activated at low temperatures to eliminate a significant fraction of the surface ligands via facile decomposition of *t*-BuNH_2_ into volatile species, such as isobutylene and NH_3_, without inducing nanoparticle sintering.

At a mild pretreatment temperature of 250 °C, ca. 25% of the total CO binding sites are made accessible on the ligand exchanged *t*-BuNH_2_–MoC_1–*x*_/C catalyst, as assessed by CO chemisorption experiments. This is compared to a complete lack of binding site availability for the as-prepared catalyst with the native oleylamine ligands. The catalytic consequence of low-temperature ligand removal was explored in the CO_2_ hydrogenation reaction. After a mild thermal pretreatment, the *t*-BuNH_2_ exchanged MoC_1–*x*_ catalyst exhibited CO and CH_4_ selectivities like those observed after a high-temperature pretreatment of both catalysts, suggesting that similar surface species were present but with a significantly lower activation temperature. We believe that this concept of mild nanoparticle catalyst activation using thermally labile ligands should be applicable to a wide range of catalytic materials by appropriately tuning the ligand binding functionality.

## Supporting Information Available

Characterization (TGA, FT-IR) of the acid-treated nanoparticles; additional ^1^H NMR and *K*_eq_ data for the UDAm ligand exchange; characterization (XRD, TEM, HR-TEM, and SAED) of the as-prepared OAm-MoC_1–*x*_ and the ligand exchanged *t*-BuNH_2_-MoC_1–*x*_ nanoparticles (PDF)
