# Supplementary material for: Activating Molybdenum Carbide Nanoparticle Catalysts under Mild Conditions Using Thermally Labile Ligands
Source: Chem Mater. 2022 Sep 22;34(19):8849–57. doi: 10.1021/acs.chemmater.2c02148 (PMC9558459; doi:10.1021/acs.chemmater.2c02148)
Supplement: Supplementary file 1 — cm2c02148_si_001.pdf [file cm2c02148_si_001.pdf]

Supporting Information

Activating Molybdenum Carbide Nanoparticle Catalysts Under Mild Conditions Using Thermally Labile Ligands

Lanja R. Karadaghi,[a] Anh T. To,[b] Susan E. Habas,[b] Frederick G. Baddour,[b] Daniel A. Ruddy,[b,*] and Richard L. Brutchey[a,*]

[a] *Department of Chemistry, University of Southern California, Los Angeles, California 90089, United States*
[b] *Catalytic Carbon Transformation and Scale-Up Center, National Renewable Energy Laboratory, Golden, Colorado 80401, United States*

E-mails: dan.ruddy@nrel.gov, brutchey@usc.edu

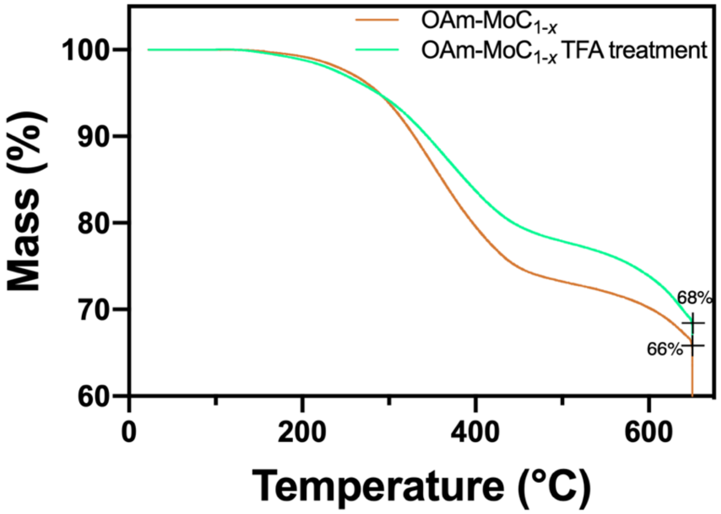

Figure S1. TGA traces comparing the as-prepared OAm-MoC_{1-x} nanoparticles and the OAm-MoC_{1-x} nanoparticles after acid treatment with trifluoroacetic acid (TFA). The final mass% of both samples after heating to 650 ˚C are nearly identical, indicating that the organic ligand content remains the same, even after the acid treatment.

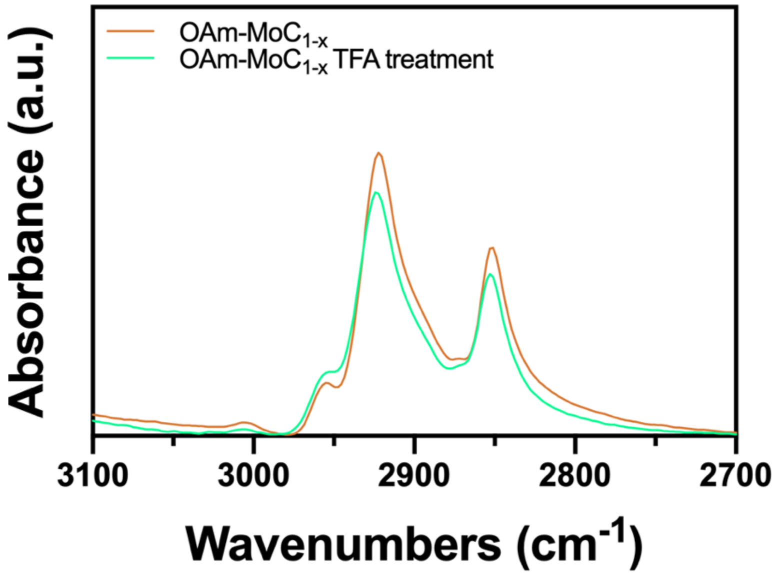

Figure S2. FT-IR spectra in the ν(C–H) stretching region of the as-prepared OAm-MoC_{1-x} nanoparticles and the OAm-MoC_{1-x} nanoparticles after acid treatment with trifluoroacetic acid (TFA). The FT-IR spectra are identical, and almost equal in intensity, indicating that little to no native oleylamine is removed from the surface of the MoC_{1-x} nanoparticles after TFA treatment.

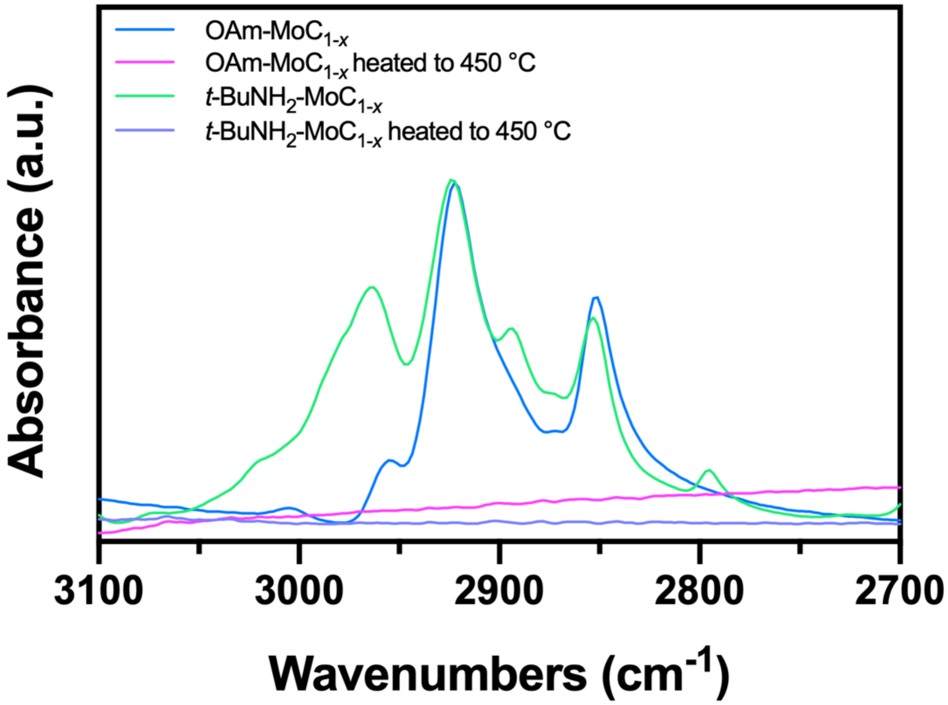

Figure S3. FT-IR spectra in the ν(C–H) stretching region of OAm-MoC_{1-x} nanoparticles, ligand exchanged *t*-$BuNH_2$-MoC_{1-x} nanoparticles, and each respective sample heated to 450 °C. After the heating to 450 °C, both samples show no stretches in this region, indicating that this thermal treatment sufficiently removes the organic ligands on the surface of these nanoparticles.

Table S1. Equilibrium constant (K_{eq}) for each titration, with increasing additions of undec-10-en-1-amine (UDAm).

μmol UDAM	K_{eq}
2.3	0.34
4.6	0.30
6.9	0.35
9.2	0.39
11.5	0.40

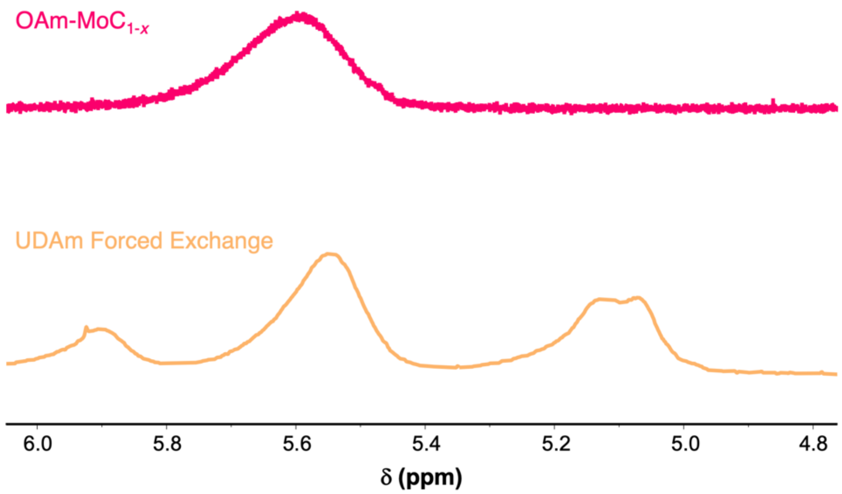

Figure S4. Solution ^{1}H NMR spectra of the alkenyl region of OAm-MoC$_{1-x}$ before (pink) and after (orange) a forced ligand exchange with undec-10-en-1-amine (UDAm). Approximately 50% of the bound OAm is displaced by UDAm, as determined through integrations with a ferrocene internal standard.

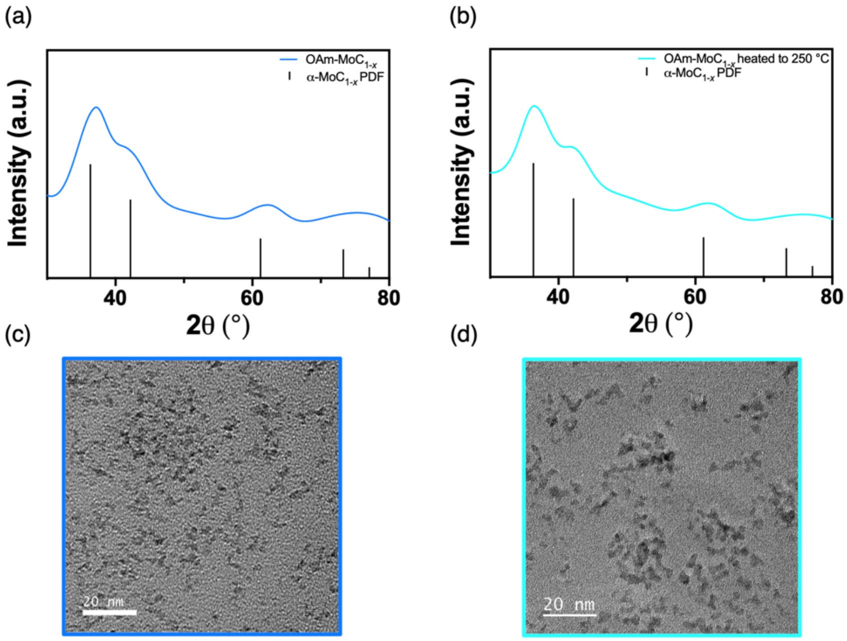

Figure S5. (a,b) XRD patterns and (c,d) TEM images of as-prepared OAm-MoC$_{1-x}$ nanoparticles before and after heating to 250 ˚C, respectively.

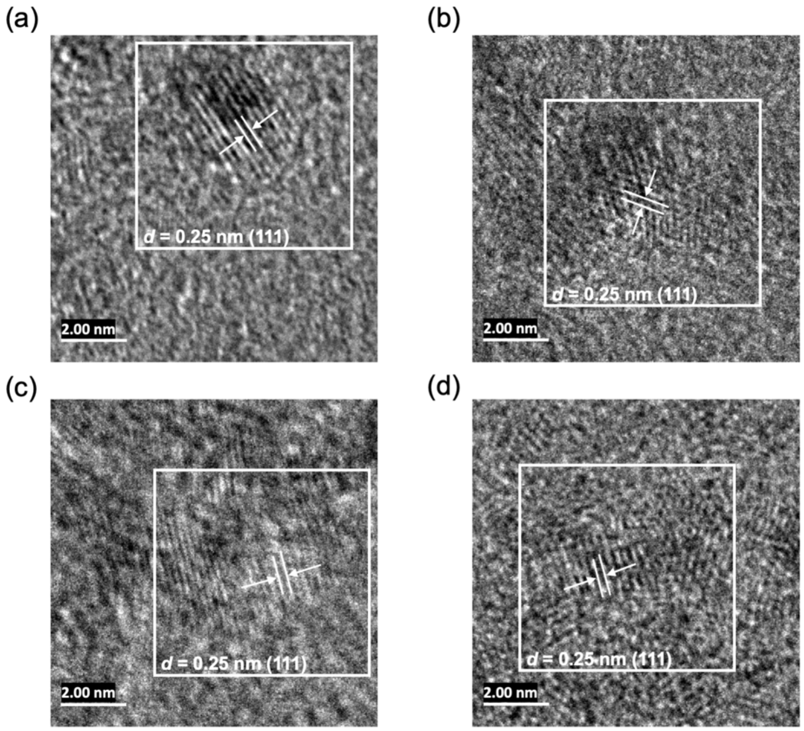

Figure S6. HR-TEM of OAm-MoC_{1-x} nanoparticles (a) before and (b) after heating to 250 °C and *t*-$BuNH_2$-MoC_{1-x} (c) before and (d) after heating to 250 °C.

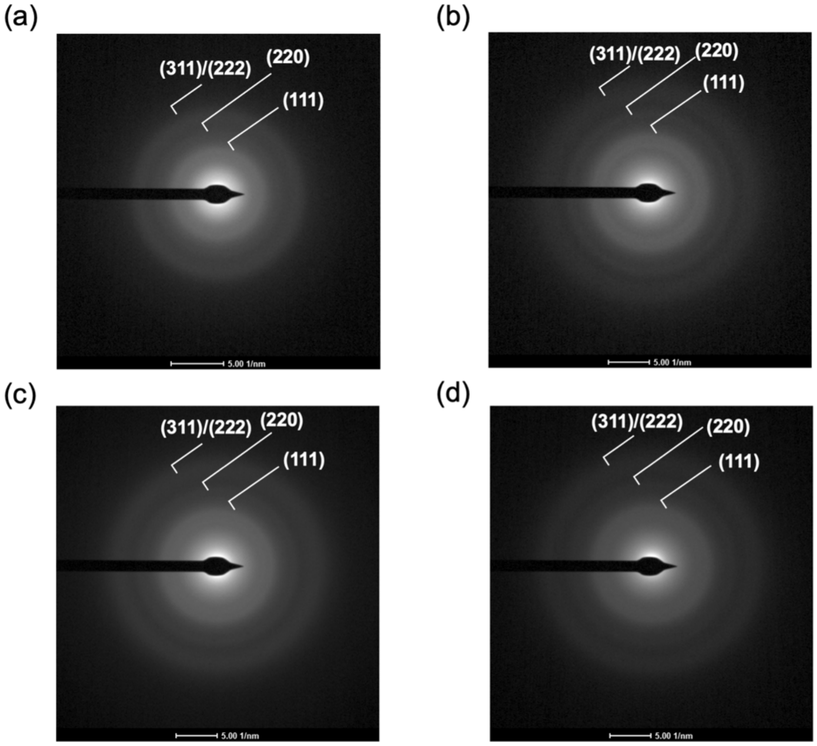

Figure S7. SAED patterns of OAm-MoC_{1-x} nanoparticles (a) before and (b) after heating to 250 °C and *t*-$BuNH_2$-MoC_{1-x} (c) before and (d) after heating to 250 °C.

Table S2. CO Chemisorption data for carbon-supported MoC_{1-x} nanoparticles after pre-treatment at 250 °C or 450 °C.

Material – Reduction Temperature	CO* site density ($umol_{CO}/g_{cat}$)
OAm-MoC_{1-x}/C – 250 °C	0.0
OAm-MoC_{1-x}/C – 450 °C	16.8
t-$BuNH_2$-MoC_{1-x}/C – 250 °C	5.1
t-$BuNH_2$-MoC_{1-x}/C – 450 °C	21.1

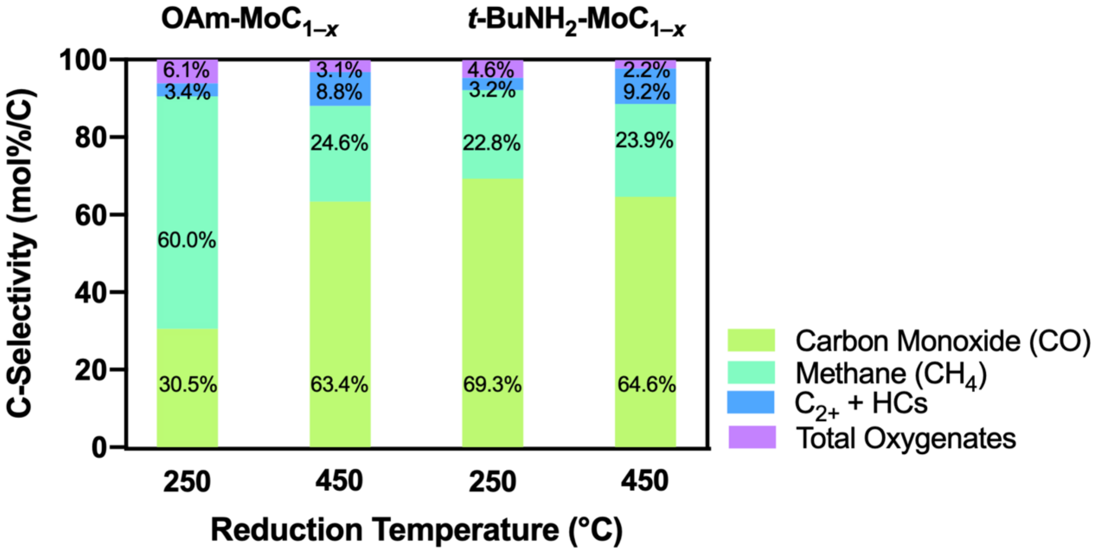

Figure S8. Product selectivities at *ca.* 10 h TOS for the carbon-supported MoC_{1-x} catalysts reduced at 250 °C and 450 °C.
